# An analytical framework for estimating aquatic species density from environmental DNA

**DOI:** 10.1002/ece3.3764

**Published:** 2018-02-25

**Authors:** Thierry Chambert, David S. Pilliod, Caren S. Goldberg, Hideyuki Doi, Teruhiko Takahara

**Affiliations:** ^1^ Ecosystem Science and Management Pennsylvania State University University Park PA USA; ^2^ CEFE Univ Montpellier CNRS Univ Paul Valéry Montpellier 3 EPHE, IRD Montpellier France; ^3^ Forest and Rangeland Ecosystem Science Center U.S. Geological Survey Boise ID USA; ^4^ School of the Environment Washington State University Pullman WA USA; ^5^ Graduate School of Simulation Studies University of Hyogo Chuo‐ku Kobe Japan; ^6^ Department of Biological Science Faculty of Life and Environmental Science Shimane University Shimane Prefecture Matsue Japan

**Keywords:** aquatic ecosystems, detection, eDNA, lentic systems, lotic systems, negative binomial model, population density, species abundance

## Abstract

Environmental DNA (eDNA) analysis of water samples is on the brink of becoming a standard monitoring method for aquatic species. This method has improved detection rates over conventional survey methods and thus has demonstrated effectiveness for estimation of site occupancy and species distribution. The frontier of eDNA applications, however, is to infer species density. Building upon previous studies, we present and assess a modeling approach that aims at inferring animal density from eDNA. The modeling combines eDNA and animal count data from a subset of sites to estimate species density (and associated uncertainties) at other sites where only eDNA data are available. As a proof of concept, we first perform a cross‐validation study using experimental data on carp in mesocosms. In these data, fish densities are known without error, which allows us to test the performance of the method with known data. We then evaluate the model using field data from a study on a stream salamander species to assess the potential of this method to work in natural settings, where density can never be known with absolute certainty. Two alternative distributions (Normal and Negative Binomial) to model variability in eDNA concentration data are assessed. Assessment based on the proof of concept data (carp) revealed that the Negative Binomial model provided much more accurate estimates than the model based on a Normal distribution, likely because eDNA data tend to be overdispersed. Greater imprecision was found when we applied the method to the field data, but the Negative Binomial model still provided useful density estimates. We call for further model development in this direction, as well as further research targeted at sampling design optimization. It will be important to assess these approaches on a broad range of study systems.

## 1 INTRODUCTION

Assessing status and trends of wild populations and communities is a challenging endeavor, especially for species that are difficult to detect (Williams, Nichols, & Conroy, 2002). The use of environmental DNA (eDNA) to detect elusive species has been a revolution in this regard (Dougherty et al., 2016; Hunter et al., 2015; Rees et al., 2014; Smart, Tingley, Weeks, van Rooyen, & McCarthy, 2015). The application of eDNA data for estimating binary metrics (i.e., presence/absence) of species distribution is now established (Biggs et al., 2015; Eichmiller, Bajer, & Sorensen, 2014). The next frontier of eDNA applications is to estimate continuous, or at least ordinal, metrics of species density and diversity (Evans et al., 2016). Several studies have demonstrated positive correlations between metrics of animal density and eDNA concentration in different aquatic organisms, including fish (Lacoursière‐Roussel, Côté, Leclerc, & Bernatchez, 2016; Lacoursière‐Roussel, Rosabal, & Bernatchez, 2016; Takahara, Minamoto, Yamanaka, Doi, & Kawabata, 2012; Wilcox et al., 2016), amphibians (Pilliod, Goldberg, Arkle, & Waits, 2013; Thomsen et al., 2012), crustaceans (Tréguier et al., 2014), and mollusks (Goldberg, Sepulveda, Ray, Baumgardt, & Waits, 2013). These studies investigated correlations between eDNA concentration in the water and animal density or biomass, but they did not assess whether it was possible to accurately predict animal density from eDNA. Indeed, eDNA data often appear to be overdispersed, an issue that could limit our ability to use eDNA information to infer metrics of animal density or biomass (Iversen, Kielgast, & Sand‐Jensen, 2015). Overdispersion in eDNA data is likely due to several factors, including [but not limited to] variation in individual shedding rates (Klymus, Richter, Chapman, & Paukert, 2015; Maruyama, Nakamura, Yamanaka, Kondoh, & Minamoto, 2014), uneven distribution of animals in the environment (Lacoursière‐Roussel, Côté, et al., 2016; Lacoursière‐Roussel, Rosabal, et al., 2016; Laramie, Pilliod, & Goldberg, 2015; Pilliod et al., 2013; Yamamoto et al., 2016), water and environmental disturbance regime (e.g., local water flow; Barnes & Turner, 2016), as well as sampling methods and environmental conditions (Goldberg, Pilliod, Arkle, & Waits, 2011; Lacoursière‐Roussel, Côté, et al., 2016; Lacoursière‐Roussel, Rosabal, et al., 2016; Pilliod, Goldberg, Arkle, & Waits, 2014).

Making the transition to estimation of animal density from eDNA concentration data requires (1) the development of adequate statistical models that account for issues inherent to eDNA studies (e.g., overdispersion) and (2) empirical validation of these models. These are the two novel elements that we provide in this article, which represents an important first step in this direction. First, we present a statistical modeling approach for the estimation of animal density for a number of sites from two sources of data: (1) “eDNA‐only data*”*: eDNA quantitative data, *without* any other type of information about animal density, available for all sampled sites; and (2) “dual data*”*: eDNA quantitative data, *associated with* reliable animal density estimates or metrics, available from a subset of sites. Our approach uses the information on the relationship between eDNA concentration and animal density, contained in the *dual data*, to infer animal density for all other sites, from the *eDNA‐only data*. Instead of relying on the *post hoc* extrapolation of a linear regression linking eDNA and density data, we provide a likelihood‐based method that combines all data in a one‐step analysis and directly provides estimates of animal density and the associated uncertainty (i.e., *SE* and 95% C.I.). After introducing the method, we assess its performance using experimental data from a controlled study on common carp (*Cyprinus carpio*). In this proof of concept study, the model was implemented on eDNA data collected in mesocosms where the number of carps was perfectly known, thus providing an ideal situation to evaluate the method's accuracy under controlled conditions. Finally, we implemented the approach on field data from a study on Idaho giant salamanders (*Dicamptodon aterrimus*) to assess its potential to work in natural settings.

## 2 MODEL DESCRIPTION

For the model described here, we consider that *K*_*i*_ eDNA water samples, obtained from either spatial or temporal replicates, are collected at each one of a total of *I* sites for which we want to infer animal density. The eDNA data are denoted *w*_*ik*_ for each site *i* = {1, … *I*} and each sampling replicate *k* = {1, … *K*_*i*_}. Data *w*_*ik*_ must be a quantitative metric of eDNA concentration (e.g., ng/l or DNA copies per ml). Ideally, the *K*_*i*_ samples for a site *i* are extracted from spatially replicated water samples pooled together and homogenized. Water replicates would typically be collected at selected locations inside the sampling unit (site; e.g., pond, wetland, section of river), at a single time, thus ensuring population closure. For diffusion‐limited lentic waters where eDNA concentrations are likely to reflect space use of the target species (e.g., Eichmiller et al., 2014), the sampling unit should be carefully identified and sampling replicates collected identically over the area of inference. Such a “snapshot” sampling design is probably more reliable than using temporally separated replicates, given that temporal variability in environmental conditions can affect eDNA. Moreover, if collection of eDNA replicates is spread across a relatively long period of time (e.g., several weeks), we are more likely to violate the assumption of closure of the target population, as birth, death, immigration, or emigration might occur between sampling occasions. In such a case, the *population density* we are trying to estimate would not be meaningful. In addition to the collection of eDNA, “traditional” surveys (e.g., repeated visual counts, trapping) must be conducted at a subset *J < I* sites to provide accurate and reliable estimates of animal density *D*_*j*_ for each site *j* = {1, …*J*}. We will refer to (1) the (*I*‐*J*) sites where only eDNA are available (i.e., uninformed animal density) as *eDNA‐only* sites and (2) the subset of *J* sites, randomly selected among the *I* sites, where both eDNA and animal density data are available, as *dual data* sites. The *dual data* sites inform the relationship between eDNA concentration and animal density and thus allow inference about animal density *D*_*j*_ at the *eDNA‐only* sites. Our results (below) suggest that very few *dual data* sites (e.g., *J* = 3–5) are necessary for the model to work properly and provide accurate estimates when the relationship between eDNA concentration and animal density is constant across sampled sites (i.e., over space and time).

The goal is to infer unknown animal density *D*_*j*_ for all sites *i* ≠ *j* from the data *w*_*ik*_ and *D*_*j*_. This can be achieved by modeling, for each site *i*, the probabilistic distribution of eDNA concentration *w*_*ik*_ as a function *f* (*D*_*i*_) of local animal density *D*_*j*_, which is known (or at least estimated) for any site *j* but totally unknown for any site *i* ≠ *j*. This model can be written in a very general fashion as:

(1) $$w_{\mathrm{ik}}\;∼\;Distr\left(f\left(D_{i}\right),\;\theta \right),$$

where *Distr* simply denotes any probabilistic distribution and **θ** represents a vector of parameters relevant to this distribution. There are three different types of model parameters here: (1) parameter(s) (**β**) describing the relationship *f*() between animal density *D*_*i*_ and the expected value of the eDNA metric *E*(*w*_*ik*_) (in the models discussed below we model this as a simple linear relationship, as *E*(*w*_*ik*_) = β_0_ × *D*_*i*_, where parameter β_0_ is the coefficient characterizing the relationship between animal density (*D*_*i*_) and the expected concentration of eDNA *E*(*w*_*ik*_)); (2) parameter(s) (**θ**) that are specific to the probabilistic distribution chosen and thus describe variability of the realized eDNA data *w*_*ik*_ around the theoretic expected value *E*(*w*_*ik*_); and (3) the unknown values of animal density (***D***_***i***_) for any site *i* ≠ *j*.

Here, we consider two different distributions to model the data *w*_*ik*_: (1) a Normal distribution that assumes that deviations of realized values *w*_*ik*_ from *E* (*w*_*ik*_) are normally distributed; and (2) a Negative Binomial distribution that allows for larger dispersion in the realized values *w*_*ik*_. To further investigate dispersion issues, we also implemented a model based on the Poisson distribution, which is a special case of the Negative Binomial distribution (see Appendix S1).

The Normal model can be written as follows:

(2) $$w_{\mathrm{ik}}\;∼\text{Normal}\left(\mu_{i},\;\sigma^{2}\right),$$

where μ_*i*_ = β_0_ × *D*_*i*_ is the expected value of *w*_*ik*_ at site *i* and σ^2^ represents across‐replicates variance in values *w*_*ik*_. The corresponding likelihood formulation is as follows:

(3) $$\prod_{i=1}^{I}\prod_{k=1}^{K}{\left(2\pi \sigma^{2}\right)}^{-\frac{1}{2}}\cdot \exp\left(-\frac{1}{2}\cdot \frac{{\left(w_{\mathrm{ik}}-\;\mu_{i}\right)}^{2}}{\sigma^{2}}\right)$$

The Negative Binomial model is written as follow:

(4) $$w_{\mathrm{ik}}\;∼\;\text{Negative Binomial}\left(\mu_{i}\;,\;r\right),$$

where μ_*i*_ = β_0_ × *D*_*i*_ is the expected value of *w*_*ik*_ at site *i* and *r* is the dispersion parameter, sometimes referred to as the “target number of successful trials” in the traditional description of the negative binomial distribution. Parameter *r* quantifies across‐replicates variability and overdispersion in values *w*_*ik*_. The likelihood formulation for this model is as follows:

(5) $$\prod_{i=1}^{I}\prod_{k=1}^{K}\frac{\Gamma \left(w_{\mathrm{ik}}+r\right)}{w_{\mathrm{ik}}!\cdot \Gamma \left(r\right)}\cdot {p_{i}}^{r}\cdot {\left(1-p_{i}\right)}^{w_{\mathrm{ik}}},$$

where, *p*_*i*_ = $\frac{r}{\mu_{i}+r}$, which also referred to as the probability of trial success of the negative binomial process. For the Negative Binomial model, data inputs must be integers. Ideally, eDNA concentration is quantified as a number of DNA copies, which does not require any transformation, as in our carp example. Otherwise, eDNA values must first be transformed as integers, like we did in our salamander example.

## 3 APPLICATION

### 3.1 Material and methods

#### 3.1.1 Proof of concept data

As a proof of concept, we applied the method on an experimental dataset on common carp, where fish density was known without error. This experiment consisted in a total of *I* = 11 non‐empty mesocosms and *K*_*i*_ = 3 eDNA sampling replicates per mesocosm (site), taken at 1‐week intervals. Here, temporal replicates were used instead of the “snapshot” spatial replicate design recommended above, but because it was a controlled experiment, variability in environmental conditions was not an issue. For full details on this case study, see Doi et al. (2015).

Model performance was quantified by the root mean squared error and 95% C.I. realized coverage of repeated animal density estimates ($\hat{D_{i}}$) provided by the model in a cross‐validation study. We used an exhaustive leave‐*p‐*out cross‐validation approach where *p* represents the number of sites for which animal density was assumed unknown (i.e., *eDNA‐only* sites) and thus used for validation. The number *J* = *I*–*p* of *dual data* sites represents the subset of data used for model calibration. We assessed the model for four different scenarios of model calibration: *I*‐*p* = {2, 3, 4, 5} *dual data* sites (*I* = 11; hence *p* = {9, 8, 7, 6}). Cross‐validations were exhaustive: For each scenario, the learning or testing process was done for all possible ways $C_{I}^{p}$ of subdividing the original dataset. For instance, for *I* = 11, with *p* = 8, a total of $C_{I}^{p}$= 165 validation repetitions were performed. For each individual repetition, we proceeded as follows: (1) *J* sites, for which we kept both eDNA and density data, were used as *dual data* sites, and *p* sites, for which only eDNA data were kept, were used as *eDNA‐only* sites for the analysis; (2) model outputs provided density estimates ${\hat{D}}_{i,i\neq j}$ for a total of *p* = *I‐J eDNA‐only* sites; (3) the *p* estimates ${\hat{D}}_{i\neq j}$ obtained as model's outputs were compared to the “known” density values that were left out of the analysis. We also calculated the error (ε_*i*_ = ${\hat{D}}_{i}$ ‐*D*_*i*_) and recorded whether the “known” value was included in the 95% confidence interval (C.I.). This process was repeated $C_{I}^{p}$ times, for all possible unique combinations. From these cross‐validation results, we then derived two summary measures to assess model performance. First, we calculated the root mean squared error (RMSE), across all cross‐validation repetitions. The RMSE is a measure of total error, combining bias and systematic error (variance, imprecision). Second, we calculated the realized coverage of the 95% C.I., across all cross‐validation repetitions, as the proportion of time that the known animal density value (*D*_*i*_) was included in the 95% C.I. produced by the model output (${\hat{D}}_{i}\pm SE$). For a well‐behaved estimator procedure, the realized coverage should be very close to 0.95.

#### 3.1.2 Field dataset

To evaluate the potential applicability of the method in natural settings, we applied the model to data obtained from a stream salamander survey performed in summer 2011 in the South Fork Salmon River Sub‐basin, Idaho, USA (Pilliod et al., 2013). During this field study, eDNA was collected and quantified at the downstream edge of a survey reach just prior to salamander sampling in five streams (see Pilliod et al., 2013 for details). Idaho giant salamanders were surveyed using single‐pass electrofishing in a 500‐m stream reach (defined as a site) of each stream and salamander density was then estimated as the number individuals captured divided by the area searched per stream. The 500‐m reach was randomly selected and predefined as the spatial sampling unit or site. While the rate of eDNA loss per distance of stream is unknown, it has been estimated to occur on the scale of 100–200 m (Wilcox et al., 2016) to as much as 9 km (Deiner & Altermatt, 2014). Here, we used the sites from the Pilliod et al. (2013) study that were sampled with electrofishing, not those that were only sampled with the kick‐net technique (see Pilliod et al., 2013) because this latter method did not provide reliable metrics of salamander densities. On the other hand, electrofishing has been shown to be one of the most reliable field sampling methods for larval and paedomorphic life stages of this species (Cossel, Gaige, & Sauder, 2012; Pilliod et al., 2013). The environmental characteristics (see Table S1) of the five sites we used were not significantly different than those of the other sites in terms of stream flow (sites used: $\overset{¯}{x}$ = 1.0 m^3^/s; other sites: $\overset{¯}{x}$ = 0.9 m^3^/s), temperature (sites used: $\overset{¯}{x}$ = 11.5°C; other sites: $\overset{¯}{x}$ = 9.7°C), and depth (sites used: $\overset{¯}{x}$ = 25.2 cm; other sites: $\overset{¯}{x}$ = 21.4 cm). Only the stream wet width differed significantly (sites used: $\overset{¯}{x}$= 491.8 cm; other sites: $\overset{¯}{x}$= 332.9 cm). Nonpaedomorphic adult salamanders, which are terrestrial, were never captured during electrofishing surveys, and thus were assumed to contribute little to no eDNA to the water. The whole dataset consisted of *I* = 5 sites (i.e., a 500‐m upstream stretch in five different streams) and the number of per‐site water sampling replicates (*K*_*i*_) varied from 3 to 12. For two sites *K*_*i*_ = 3, for two others *K*_*i*_ = 9 and for one site *K*_*i*_ = 12. The data for the two sites with nine samples actually consisted of three replicated samples from each of three slightly different methods of water collection (GrabFilter, GrabHold, Instream; see Pilliod et al., 2013). We were confident in using all nine samples together because (1) the difference between methods only concerned water collection and storage (not eDNA processing and analysis), and (2) previous investigation had shown no evidence to suggest differences in the amount of eDNA captured by each method for these samples (Pilliod et al., 2013, pp 1126).

To assess models’ performance on this dataset, we used the same cross‐validation approach as described above, but with the caveat that salamander densities were not known perfectly. Because densities were estimated from a field sampling technique (electrofishing) that cannot provide perfect detection of individuals, we acknowledge that there is inherent uncertainty in the density data that feed the model, which likely affects the accuracy of our estimator. Despite this caveat, we think it was useful to assess the model with field data obtained from typical methods used by field biologists. These imperfect density measures still provide useful relative measures of true density, which we believe capture consistent differences among the sampled sites.

### 3.2 Results

**Figure 1:**
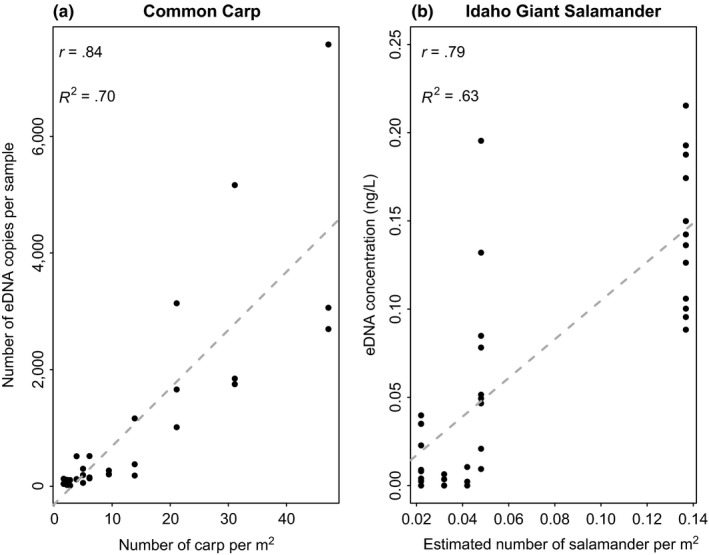
Linear regression between eDNA concentration data and measures of animal density for the two datasets. The correlation (*r*) and proportion of explained variance (*R* ^2^) values are both shown on each graph. (a) Common carp dataset: number of eDNA copies quantified through droplet digital PCR across values of carp density (carps/m^2^). (b) Idaho giant salamander dataset: concentration (ng/l) of eDNA quantified through qPCR across values of salamander density (salamanders/m^2^). We note that the absolute SD among sampling replicates tends to increase with larger values of DNA concentration

Before running analyses with our new modeling approach, we looked at correlations between values of eDNA concentration and animal density, to get a sense of the “quality” of information contained in the dataset. Correlation was high for both the proof of concept (*r* = .84, Figure 1a) and the field datasets (*r* = .79, Figure 1b). We also quantified the degree of overdispersion in eDNA, using average values of *per‐site* variance‐to‐mean ratio (VMR), with VMR > 1 indicating overdispersion and VMR < 1 indicating underdispersion. This information was especially useful to compare performances of the different models considered. We found a high degree of overdispersion in the carp eDNA data, with the *per‐site* VMR averaging 182.2. Here, no data transformation was applied because the original eDNA data were quantified as a *number of DNA copies per ml*, which are already integer values.

For the salamander data, the *per‐site* VMR averaged 0.49, which is much lower than the VMR value observed for the proof of concept dataset. To apply the Negative Binomial model, values of eDNA concentration (initially expressed in ng/l) were transformed as integers, after having been scaled to the level of precision with which DNA concentration can be measured (see details in Appendix S2).

#### 3.2.1 Proof of concept data

**Figure 2:**
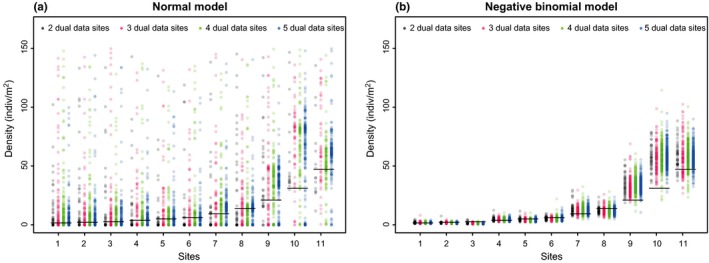
Results of the cross‐validation study from the common carp dataset (mesocosm experiment). Each point represents the density estimate obtained from (a) the Normal model and (b) the Negative Binomial model, for cases where fish density was known for 2 (black), 3 (red), 4 (green), or 5 (blue) sites (i.e., *dual data* sites). The horizontal black dashes represent the known values of animal density

Results from the cross‐validation study clearly indicated that the Normal distribution was inappropriate to model the carp eDNA data and make inference about carp density. With the Normal model, estimators of carp density were inaccurate (Table 1, Figure 2a), with a RMSE > 1310 in the best case scenario, which is to be compared to the range of values of true carp density that lies between 1.7 and 47.2 indiv/m^2^ (the RMSE was > 100 times larger). We can also see from Figure 2a that, in many cases, the estimators of carp density were biased high. Moreover, the realized coverage of the 95% C.I. was poor, between 0.37 and 0.44. On the other hand, the Negative Binomial model provided more accurate estimates of carp density (Table 1, Figure 2b, Table S2). The RMSE was between 10 and 12, which seems reasonable. The coverage of the 95% C.I. was also good, falling between 0.95 and 1.00 for the four scenarios considered (i.e., different number of *dual data* sites *I*‐*p* = {2, 3, 4, 5}). Increasing the number of *dual data* sites from two to five does not seem to improve estimator accuracy or coverage level very much. This suggests that small sample sizes of *dual data* sites might be appropriate to achieve good estimator properties, but it is important to keep in mind that these small numbers still represent fairly large proportions of the total number of sites (between 18% and 45%).We also assessed the ability of the Negative Binomial model at providing relative rankings of site density. We found rates of correct relative rank assignment of 76.2%, 76.8%, 78.9%, and 81.5% for scenarios with 2, 3, 4, and 5 dual data sites, respectively. Estimate of the dispersion parameter *r* was 0.94 (*SE* = 0.412), a value in accordance with the highly overdispersed nature of these data (note: the more overdispersion, the smallest the values of *r*; we consider no overdispersion as *r*→∞). As a consequence of this high degree of overdispersion, the Poisson model (i.e., equivalent of the Negative Binomial but without overdispersion) showed much poorer performance, in terms of C.I. coverage, than the Negative Binomial (Appendix S1).

**Table 1:** Summary results of analyses for both dataset. The root mean squared error (RMSE) and the 95% C.I. coverage are shown. See Figures 2 and 3 for a detailed plot of individual estimates for the different scenarios assessed

	Normal model		Negative binomial model	
	RMSE	Coverage	RMSE	Coverage
Proof of concept analysis: common carp dataset				
2 dual data sites	1,310	0.39	11	1.00
3 dual data sites	4,337	0.39	11	0.97
4 dual data sites	15,403	0.37	12	0.97
5 dual data sites	44,340	0.44	10	0.95
Field data analysis: Idaho giant salamander dataset				
2 dual data sites	0.06	0.82	0.03	1.00
3 dual data sites	0.08	0.84	0.02	1.00

#### 3.2.2 Field dataset

**Figure 3:**
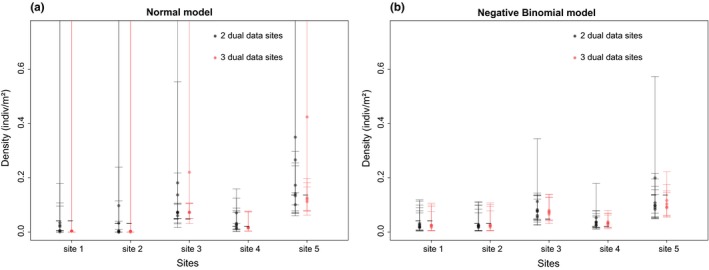
Results of the cross‐validation study from the Idaho giant salamander dataset (from electrofishing sampling of wild populations). Each point represents the density estimate obtained from the (a) Normal and (b) Negative Binomial models, for cases where salamander density was “known” for 2 (black) or 3 (red) sites (i.e., *dual data* sites). The associated error bars, representing the 95% C.I for each individual estimate, are also showed here. The horizontal black dashes represent the relative values of animal density obtained from field data. For the Normal model, the higher limit of the 95% C.I. is not visible for a few scenarios because it out of scale (upper limits between 67.3 and 6,778). This is of course a clear indication of the poor precision of this model

Similarly to the proof of concept dataset, we found the Negative Binomial model to perform better than the Normal model (Table 1, Figure 3). Here, only 2 and 3 *dual data* sites were used to inform the eDNA/salamander density relationship, but it represented 40% and 60% of all the sites (*I* = 5), respectively. The realized coverage was 1.00 for both scenarios assessed. Typically, this would reflect low precision associated with individual estimates (i.e., large 95% C.I.'s), but, as we can see on Figure 3b, individual 95% C.I.'s do not appear overly large. Almost all individual 95% C.I.'s are of similar extent and they cover a reasonable range of values. The RMSE, in both case scenarios, were not large (RMSE = {0.02, 0.03}, Table 1) relative to the range of estimated *D*_*i*_ values ($\hat{D_{i}}\in$ {0.022, 0.137}, $\hat{D_{i}}$. = 0.056). The Negative Binomial model also showed good performance in terms of relative ranking of site's density (76.7% and 90% correct ranking, for scenario with 2 and 3 dual data sites, respectively). The Normal model did not perform nearly as well, showing (1) higher total error (RMSE), (2) larger biases, (3) lower precision (very large individual 95% C.I.'s; Figure 3a), and (4) lower coverage. In accordance with the low VMR values (0.49) of this dataset, estimates of the dispersion parameter *r* were very large ($\hat{r}$ = 44,914). We were thus not surprised to see the Poisson mol perform as well as the Negative Binomial (Appendix S1).

## 4 DISCUSSION

We provided a model framework to estimate animal density using eDNA data in combination with known animal density values obtained for a subset of sampled sites. Cross‐validation analyses, performed using a dataset for which fish densities were known with certainty, revealed that this approach can work well when the correlation between eDNA data and animal density values is high. Cross‐sample variability of eDNA concentration was best modeled with a Negative Binomial distribution, as found in a previous study (Furlan, Gleeson, Hardy, & Duncan, 2016). Although the results from the field dataset must be interpreted with caution, because salamander density values bear substantial uncertainty, they also supported these findings. Indeed, here too, the Negative Binomial clearly outperformed the Normal model, and it provided useful estimates of salamander densities, although not as accurate as in the experimental data analysis. These findings highlight the inadequacy of the Normal distribution to model variability in the link between eDNA and animal density. This strongly suggests that *ad hoc* extrapolations based on simple linear regression, which assume Normal errors, should be avoided. Generalized regressions, as well as maximum likelihood and Bayesian approaches, based on negative binomial (sometimes Poisson) distributions are more appropriate (Furlan et al., 2016).

The main limit of our method, currently, lies in the fact that it does not explicitly account for uncertainty in animal density values $\hat{D_{j}}$. Uncertainty was not an issue with the carp data because the number of animals in each mesocosm was known. These data were ideal to assess model performance as proof of concept scenario, but they may not reflect field levels of eDNA in the environment. Using survey estimates of animal density (from the traditional surveys) as data points, without consideration of the associated uncertainty, can create biases and erroneously inflate our confidence in the eDNA‐based estimates of animal density. We therefore emphasize that it will be important to pursue model developments of this approach in order to (1) add a detection‐based process for density estimation in the modeling frame and (2) explicitly incorporate uncertainty of $\hat{D_{i}}$ values when modeling the eDNA‐density relationship (i.e., the model presented here). Both goals could be achieved by modeling these different processes in a hierarchical framework (Royle & Dorazio, 2008) using, for instance, Bayesian inferential tools (Link & Barker, 2009). More specifically, it could be performed within a MCMC routine by (1) explicitly incorporating the estimation procedure of ${\hat{D}}_{j}$ (e.g., mark–capture, N‐mixture models, etc.) in a Bayesian model and (2) sampling the posterior distribution of parameters ${\hat{D}}_{j}$, instead of using point estimates.

Another important area that deserves discussion and further research is sampling design. As with any estimator, the key to obtaining reliable estimates relies on our ability to obtain representative samples and characterize the probabilistic distribution (e.g., variability) of the *biological* and *sampling processes* involved in the data generating mechanisms. Here, these mechanisms can be summarized hierarchically as consisting of (1) a latent process relating true (unknown) animal density (*D*_*i*_) to the expected (average) concentration of eDNA [*E*(*w*_*ij*_)] in the environment and (2) another unknown process for the variability in the actual values of eDNA concentration collected and quantified through PCR [*w*_*ij*_|*E*(*w*_*ij*_)]. The first process combines elements of both the biological and environmental processes (Barnes & Turner, 2016) that determine (1) the rate of DNA shedding and production by the focal organisms, (2) how this DNA will be transported and distributed in the environment (spatial aspects), and (3) how long it will persist in the environment (temporal aspects). Altogether, these elements will determine how much DNA from the focal species is *available* to sampling in the environment. This is formalized in our model by the function *f (D*_*i*_ *)* which ultimately determines the expected value of the eDNA in our samples for a given local animal density (*E*(*w*_*ij*_)|*D*_*i*_). The investigator cannot control any of these elements, but the timing and spatial design of water sample collection will affect the amount of total *available* eDNA exposed to sampling. It is essential for the application of the method that the relationship between density and eDNA available to sampling remains consistent across sampling sites for which inference is to be made. The second process combines elements of sampling design and laboratory processing, which can be regrouped under the term “*sampling process,*” determining the concentrations of eDNA actually measured (*w*_*ij*_), for a given amount that was *available* for sampling. This process is formalized in our model by the choice of specific distributions: *w*_*ij*_| *E*(*w*_*ij*_)~*Distr*(*E*(*w*_*ij*_), **θ**). The accuracy with which we can characterize this distribution and estimate model's parameters will strongly be affected by the sampling design chosen. We therefore encourage endeavors that focus on improving eDNA sampling (Goldberg et al., 2016). Important study design issues concern the timing and location of sampling, as well as the number of water sample replicates and the volume of water collected. Timing of eDNA sampling should be chosen in relation to the species’ life cycle and in consideration of the assumption of population closure (Williams et al., 2002). Indeed, as with any monitoring effort, sampling must occur when all individuals from the focal population are available for detection. It is also important that the independent density data (e.g., animal counts) used for model calibration be collected at virtually the same time as the eDNA data.

The exact location at which water samples should be collected is also of prime importance for this method. First, it is important to clearly define the sampling unit to which inference is to be made. In lentic systems that are naturally delimited in space, such as pond or lakes, the site and local population sampled are usually easily defined. However, to accurately sample such an enclosed space, the water from which eDNA is to be extracted must be representative of the entire sampling unit (e.g., whole pond). Individual spatial replicates, which would be analyzed separately for DNA, can be used directly as sampling replicates. However, because animal space use, and thus target DNA distribution, is likely to be heterogeneous within the unit, such a design is likely to inflate variability in eDNA measurements. Instead, we recommend using a sampling design where each sampling replicate is obtained from several spatial replicates covering the entire unit of interest. The latter could be pooled and homogenized before proceeding to water filtering and DNA amplification. Repeating this procedure for each individual sampling replicates (i.e., repeat *K*_*i*_ times) will provide the most representative sample for DNA concentration and density. As an alternative, but less desirable, one could resample a homogenized bulk of water to obtain the desired number of sampling replicates. In open lotic systems such as streams and rivers, limits of spatial inference might be less obvious. Moreover, eDNA transport distance, which is affected by water flow, adsorption, and degradation, is a critical, but currently poorly understood component in lotic systems (Jane et al., 2015; Wilcox et al., 2016). Animal distributions and movements may also influence eDNA concentrations and transport along a stream with unknown consequences for abundance estimation (e.g., input from an upstream population, local eDNA unavailable due to downstream export). If the system is open, this will cause issues similar to violation of the (temporal) closure assumption discussed above (Williams et al., 2002). In our salamander example, we know that salamander movements are limited relative to the scale of sampling (J.O. Cossel, unpublished data), but transport of eDNA downstream could still be an issue (Pilliod et al., 2014).

The quantity of water filtered for each sample will affect the likelihood of obtaining outlier values of eDNA concentration *W*_*ik*_, which are due to potential aggregation of eDNA distribution in the environment (Barnes & Turner, 2016; Lacoursière‐Roussel, Côté, et al., 2016; Lacoursière‐Roussel, Rosabal, et al., 2016). Therefore, the more water is filtered, the more likely we are to dilute this aggregation pattern and tend toward the local average concentration of eDNA in our individual samples. Sampling replication is crucial to better characterize the relationship$\;f\left(D_{i}\right)$ and the pattern of variability in individual samples. Given the overdispersed nature of quantitative eDNA data, the consideration of the adequate number of spatial replicates of water samples is even more important. Similarly, it will be important to carefully consider the number of technical replicates for each individual water sample *k* processed in the laboratory to provide values of eDNA concentration. Finally, it is important to mention that the consistency and sensitivity of DNA analysis methods will influence the precision of estimates.

Besides sampling issues concerned with the eDNA component, it will also be important to consider how many dual data sites are required and what sites should be targeted to accurately inform the eDNA–animal density relationship. The selection of dual data sites should be random, but it is also important to consider the sites’ characteristics when making these choices. Indeed, the physical and environmental characteristics (e.g., flow, discharge level, substrate, etc.) of the sites might influence the eDNA–density relationship. Therefore, a stratified sampling strategy across these characteristics would ensure representation of this variation. With enough sites, these measures could be used as covariates to further refine these modeled relationships. Methodologic work aimed at assessing and optimizing all these aspects of sampling design (eDNA and dual data sites) could also improve the reliability of the analytical method presented here.

Concerns and skepticism about using eDNA to estimate animal density have been raised (Iversen et al., 2015). The main argument of these critics relies on observations of heterogeneity in organism's individual DNA shedding rates (Klymus et al., 2015; Maruyama et al., 2014). Although we agree this is a relevant point that complicates estimation of animal density from eDNA, we emphasize that the existence of these sources of variation and uncertainties could be accommodated through appropriate statistical models (e.g., explicitly modeling sources of heterogeneity with covariates or random effects) and will not necessarily prevent density estimation. For us, the main limiting factor for this method to work appropriately in field studies concerns the level of correlation at the population level, not at the individual level, between eDNA concentration in the environment and local animal density. Strong correlations can exist despite substantial variability in individual shedding rate (Doi et al., 2015; Thomsen et al., 2012), making eDNA concentration a potentially useful source of data to estimate, or at least provides relative indices of animal density. However, we acknowledge that this method will not work in all settings (e.g., when population sizes are extremely low, Spear, Groves, Williams, & Waits, 2015). The presence of outliers will also influence the strength of existing correlations (Biggs et al., 2015; Pilliod et al., 2013), but as illustrated here, appropriate statistical distributions can be used to account for this phenomenon.

Environmental DNA methods are already showing great promise (Goldberg, Strickler, & Pilliod, 2015; Goldberg et al., 2016), and we are convinced that, over the years, they will follow a path of method developments (e.g., laboratory techniques, modeling, sampling designs), which will make them a standard tool for wildlife monitoring and ecological science. Our modeling approach represents one of the first steps to advance the difficult, but important topic of inferring animal density from eDNA data. This work builds upon previous studies (Barnes & Turner, 2016; Goldberg et al., 2013; Lacoursière‐Roussel, Côté, et al., 2016; Lacoursière‐Roussel, Rosabal, et al., 2016; Pilliod et al., 2013; Takahara, Minamoto, & Doi, 2013; Takahara et al., 2012; Wilcox et al., 2016), but moves beyond a simple *post hoc* extrapolation based on linear regressions. The next big steps in this methodological development will require: (1) further model development to accommodate varying sources of uncertainties and allow incorporation of environmental covariates, fixed, and random effects; (2) further investigation of sampling design optimization; and (3) testing of different species in natural lotic and lentic waters.

## DATA ACCESSIBILITY

Data files are all provided in Supporting information.

## AUTHOR CONTRIBUTIONS

TC conceived and developed the statistical model and methods; CG and DP conceived the salamander field sampling protocols and collected these data; CG performed the salamander eDNA analyses; TT and HD conceived the carp experiment and collected these data; HD performed the carp eDNA analyses; TC analyzed the data; TC, DP, CG, and HD led the writing of the manuscript. All authors contributed critically to the drafts and gave final approval for publication.
