# Supplementary material for: An analytical framework for estimating aquatic species density from environmental DNA
Source: Ecol Evol. 2018 Feb 25;8(6):3468–77. doi: 10.1002/ece3.3764 (PMC5869225; doi:10.1002/ece3.3764)
Supplement: Supplementary file 1 [file ECE3-8-3468-s001.pdf]

Table S1. Environmental characteristics of the study streams of the Pilliod et al. (2013) study. These data were measured at the time and location of eDNA sampling (July or August 2011). In our analyses, we only used the five sites (top table) at which sampling was done using electrofishing.
Some data were not available (NA).

Stream characteristics of the five sites (electrofished) used in the analysis

Stream	Water temperature (C)	Channel wetted width (cm)	Average water depth (cm)	Stream flow (m^3/s)
Weir	12.5	650	21	NA
Fitsum Main	11	446	25	0.8
Fitsum North Fork	12	515	28	1.3
Goat	14	413	23	0.7
Reegan	8	435	29	1.3
**MEAN**	**11.5**	**491.8**	**25.2**	**1.0**

Stream characteristics of the other sites from the Pilliod *et al.* (2013) study (not used in this analysis)

Stream	Water temperature (C)	Channel wetted width (cm)	Average water depth (cm)	Stream flow (m^3/s)
Blackmare	10	328	25	0.8
Buckhorn North Fork	6	533	36	2.1
Deadman	13	148	19	0.2
East Fork of the South Fork Salmon	8	562	29	1.8
Four Mile	12	469	29	1.2
Meadow Upstream	11	385	34	1.4
Nasty	11	236	25	0.5
Parks	9	425	31	1.5
Williams	13	93	11	0.1
Deadwood	7	403	20	0.5
East Fork Deadwood	7	495	11	0.3
Little House	NA	158	5	NA
Pole	NA	307	10	NA
Three	NA	119	15	NA
**MEAN**	**9.7**	**332.9**	**21.4**	**0.9**
