# Supplementary material for: An analytical framework for estimating aquatic species density from environmental DNA
Source: Ecol Evol. 2018 Feb 25;8(6):3468–77. doi: 10.1002/ece3.3764 (PMC5869225; doi:10.1002/ece3.3764)
Supplement: Supplementary file 2 [file ECE3-8-3468-s002.pdf]

Table S2. Estimator properties of the Negative Binomial model obtained from the cross validation study of the common carp dataset. Results are shown for the four scenarios run, respectively including 2, 3, 4 and 5 dual data sites. The expected estimator value (“Expected”) is shown, as well as the lower (LL) and upper (UL) limits of the 95% interval of the sampling distribution. Carp density is the parameter estimated by this model. The real (known) carp density is also provided.

Site	Real Density	*2 dual data sites*			*3 dual data sites*			*4 dual data sites*			*5 dual data sites*		
		Expected	LL	UL	Expected	LL	UL	Expected	LL	UL	Expected	LL	UL
site 1	1.67	2.05	1.20	4.18	1.94	1.15	3.30	1.84	1.22	3.15	1.78	1.28	2.75
site 2	2.22	2.01	1.18	2.68	1.81	1.15	3.12	1.75	1.22	2.61	1.65	1.18	2.48
site 3	2.78	1.67	0.82	4.18	1.34	0.85	2.51	1.26	0.86	1.96	1.17	0.80	1.81
site 4	3.89	4.95	3.13	8.39	4.49	2.85	7.29	4.57	3.06	6.74	4.49	3.13	6.40
site 5	5.00	4.64	2.90	7.97	4.36	2.75	6.61	4.18	2.83	5.93	4.15	2.85	6.36
site 6	6.11	5.51	3.42	9.26	5.21	3.20	8.06	5.30	3.37	8.68	5.25	3.49	8.62
site 7	9.44	13.60	7.75	28.06	12.27	6.82	18.62	12.81	7.55	20.22	12.30	7.90	18.45
site 8	13.89	12.39	8.72	19.60	12.06	6.98	20.20	12.04	7.24	19.22	11.73	7.57	16.80
site 9	21.11	34.49	21.49	50.80	32.57	21.84	56.13	34.09	21.27	49.45	33.31	23.25	47.74
site 10	31.11	57.31	37.73	78.01	56.25	36.25	80.94	58.43	32.17	80.41	57.86	42.09	78.94
site 11	47.22	58.87	40.95	82.16	57.87	37.35	92.88	57.82	32.56	78.39	56.86	40.25	76.43
