# Supplementary material for: An analytical framework for estimating aquatic species density from environmental DNA
Source: Ecol Evol. 2018 Feb 25;8(6):3468–77. doi: 10.1002/ece3.3764 (PMC5869225; doi:10.1002/ece3.3764)

**Appendix S1. Implementation of the Poisson model**

***Methods***

To further the issue of overdispersion and the benefits of using a Negative Binomial distribution, we also implemented the model using a Poisson distribution, which is an obvious null to assess overdispersion model when dealing with count data. Indeed, the Poisson distribution is a special case of the Negative Binomial distribution, where $r\overset{\to}{} \infty$. The Poisson distribution has a single parameter,$\lambda$, which corresponds to both its mean and variance. For the eDNA data [$y_{ik}$, see main text for details], the Poisson model can be written as:

$$y_{ik} \sim Poisson\left( \lambda_{i} \right),$$

where$\lambda_{i}=\beta_{0}\times D_{i}$ is the expected value of $w_{ik}$ at site $i$, as well as its across-sample variance. The likelihood is:

$$\prod_{i=1}^{I} \prod_{k=1}^{K} \frac{{\lambda_{i}}^{y_{ik}}e^{-\lambda_{i}}}{y_{ik}!} .$$

***Results***

### Proof of concept dataset (Carp data)

The Likelihood optimization of this Poisson model systematically failed when applied to the original carp data. This was directly due to the high degree of dispersion of these data (variance to mean ratio: VMR = 182.2). Therefore, we decided to apply a transformation to the data to reduce count (DNA copies) dispersion. We divided the number of DNA copies by 10 (and rounded the output), which led to a VMR of 18.1 and allowed the Poisson model’s likelihood optimization to work.

Results are shown on **figure S1**. Estimates from the Poisson model are relatively accurate and, in that regards, they are comparable to those of the Negative Binomial model. However, the 95% C.I. coverage of the Poisson model’s estimates are much poorer (between 0.53 and 0.61, **Fig. S1**) than that of the Negative Binomial model (0.99). This highlight the fact that more imprecision remains

### Field dataset (salamander data)

The Salamander dataset displayed a lower degree of dispersion (VMR = 0.49), and as a consequence, the Poisson model happened to perform equally well as the Negative Binomial (**Fig. S2**).

**Figure S1.** Results of the analysis applied to the transformed *proof of concept* Carp data: (A) Results from the Poisson model; (B) Results from the Negative Binomial Model. The data transformation consisted in dividing the number of DNA copies by 10 to reduce dispersion.


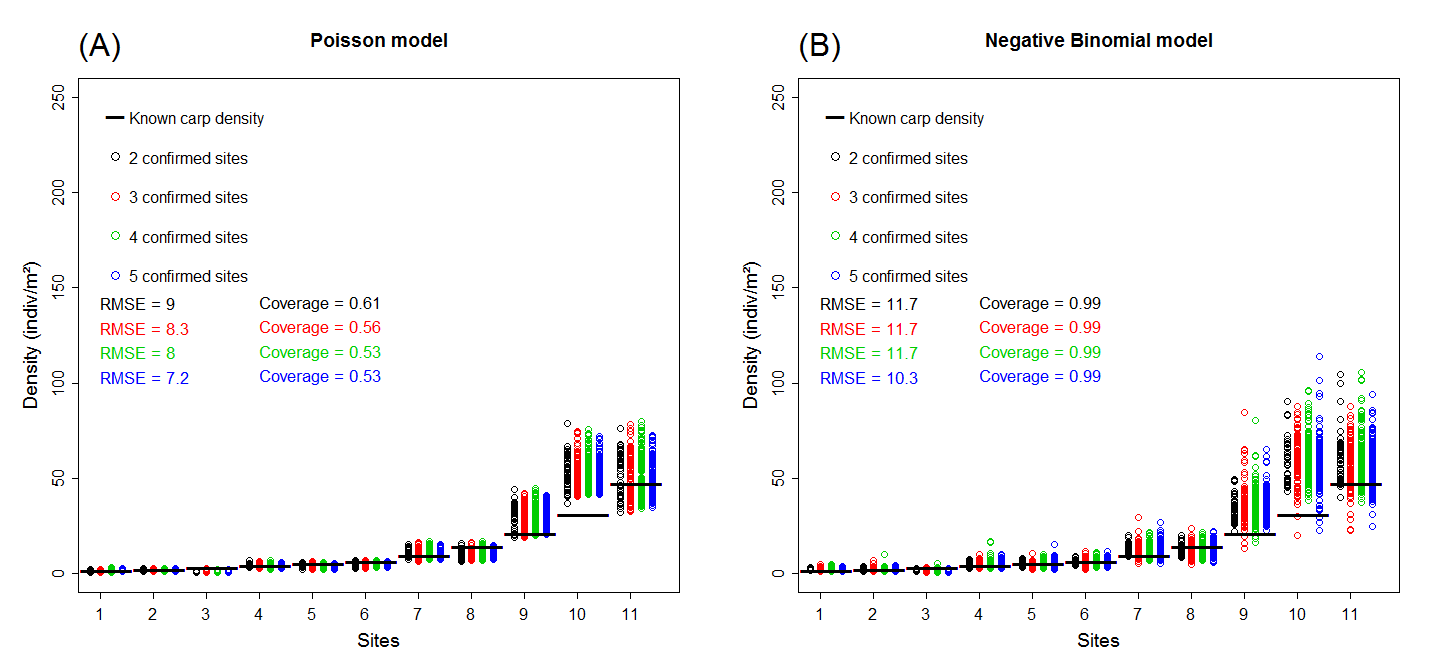


**Figure S2.** Results of the analysis applied to the field data (salamanders): (A) Results from the Poisson model; (B) Results from the Negative Binomial Model. Both model performed equally well.


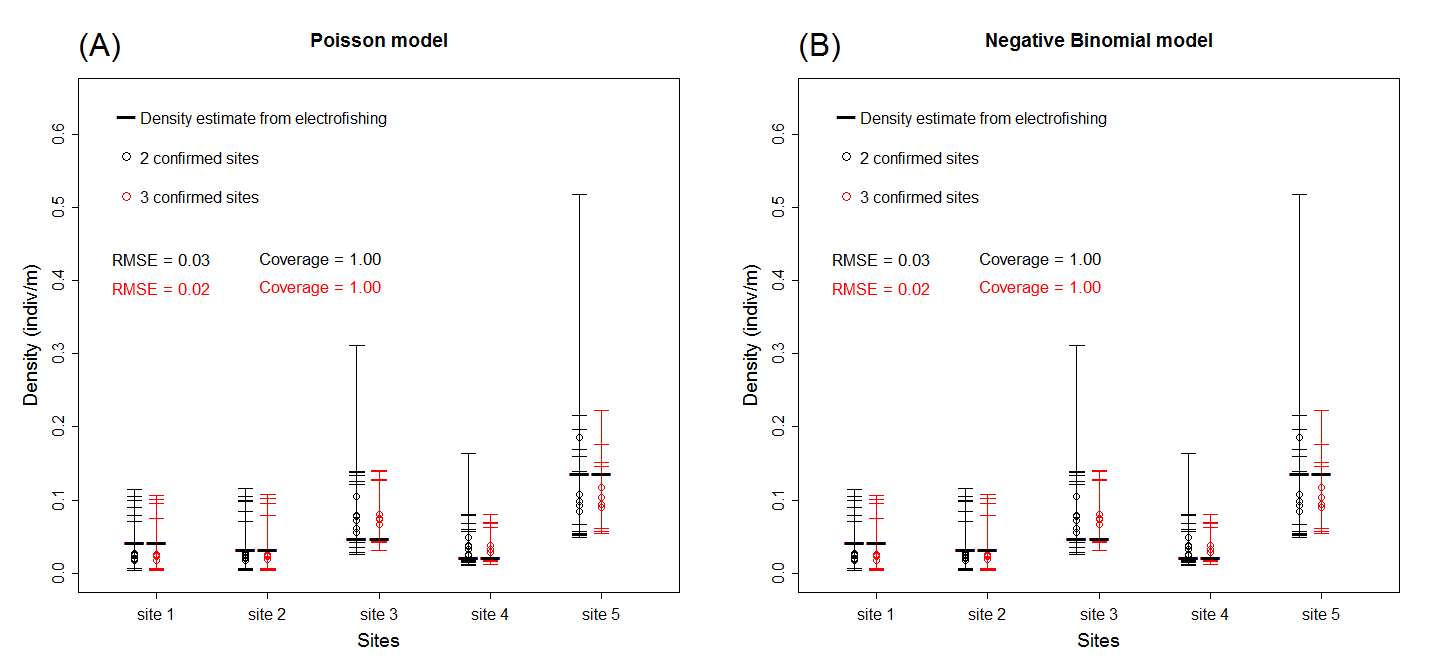

Supplement: Supplementary file 3 [file ECE3-8-3468-s003.docx]
