# Supplementary material for: An analytical framework for estimating aquatic species density from environmental DNA
Source: Ecol Evol. 2018 Feb 25;8(6):3468–77. doi: 10.1002/ece3.3764 (PMC5869225; doi:10.1002/ece3.3764)
Supplement: Supplementary file 4 [file ECE3-8-3468-s004.docx]

**Appendix S2. Transformation of eDNA concentration data as integers**

To apply a Negative Binomial distribution, we had to transform the eDNA concentration data (e.g., ng.L^-1^) as integers. In the text below, we describe the process and the rationale we use to transform the concentration data of the salamander dataset. We remind the reader that no transformation was needed for the carp data as they were initially expressed as integers (number of eDNA copies per sample). Below, we will refer to the original concentration data, for a given site *i* and a given replicate *k*, as $w_{ik}$ and to the transformed data (integer value) as$y_{ik}$.

Applying such a transformation (rounding) deserves some caution because it affects the total amount of variability contained in the data. First, because eDNA quantitative metrics such as eDNA concentration can be expressed at many different unit scales (e.g., ng.mL^-1^ vs. µg.L^-1^), the rounding of $w_{ik}$ would give very different values depending on the unit chosen, which arbitrarily affects the amount of variability retained in the transformed data $y_{ik}$. Similarly, when dealing with small values of eDNA concentration (e.g., $w_{ik}\ll$1), it will be necessary to first multiply $w_{ik}$ values by a constant *g* (hereafter, referred to as the *rounding factor*, *g*), before rounding to the unit, to capture some variability in the data; otherwise, all transformed values $y_{ik}$ would be equal to zero.

As an example, let us consider 3 samples for which eDNA concentrations were respectively measured as $\boldsymbol{w}$= {0.45001, 0.02184, 0.12376}. If we apply a *rounding factor* *g =* 100, we would obtain values {45.001, 2.184, 12.376} and after rounding to the unit, we would have $\boldsymbol{y}$ = {45, 2, 12}, with a SD of 19.7. Applying a *rounding factor* *g* = 10^5^ would give values {45001, 2184 and 12376}, with a SD of 19853. Therefore, we must seek to choose a *rounding factor* that captures a relevant level of data variability. If we use a *rounding factor* that is too small, we will artificially reduce the amount of variability captured in the transformed data$y_{ik}$, inducing a loss of information. With a *rounding factor* that is too large, we will artificially increase data overdispersion. From the example above with $\boldsymbol{w}$= {0.45001, 0.02184, 0.12376}, we could easily argue that using a rounding factor capturing precision to the fifth digit (*g* = 10^5^) might unnecessarily overdisperse the data. On the other hand, if original values were $\boldsymbol{w}$= {0.001782, 0.000867, 0.000944}, this might be appropriate, producing $\boldsymbol{y}$= {178, 87, 94} with a SD = 50.64. A *rounding factor* can take any value *g* > 0, and values *g* < 1 might actually be used if the original unit scale was very small (i.e., large $w_{ik}$ values) and the data excessively overdispersed.

A relevant level of precision to express eDNA quantities can be estimated from the data, especially if replicated qPCR measures, typically run in triplicates, are available. Indeed, the across-triplicates SD (${SD}_{w_{ik}}$), for each sample ($i,k$), provides an estimate of measurement error for each sample. We note that, when triplicate measures are run, the across-triplicates *average* value is usually used as the unique data point$w_{ik}$. To account for the magnitude of eDNA concentration $w_{ik}$ and its variability across samples ($i,k$), these measures of sampling error can be expressed as a coefficient of variation (${CV}_{w_{ik}}={SD}_{w_{ik}}/w_{ik}$). The average value of ${SD}_{w_{ik}}$ (hereafter,$\bar{SD}_{w}$), across all sites and spatial replicates *I* x *K*, provides an estimate of the overall level of dispersion (i.e., error) in $w_{ik}$ values, and can thus be used to estimate a relevant level of data precision. To account for variability in the magnitude of eDNA quantity across spatial replicates, $\bar{SD}_{w}$ is derived from values ${CV}_{w_{ik}}$ as:

$$\bar{SD}_{w}= \bar{CV}_{w}\times\bar{w}$$

where $\bar{CV}_{w}$ and $\bar{w}$ represents the average values, across all sites and spatial replicates, of ${CV}_{ik}$ and $w_{ik}$, respectively. The *rounding factor* to be used for data transformation is thus *g* =$1/ \bar{SD}_{w}$, which captures the level of precision in the original data. With this constant *g*, the averaged magnitude of error (±$\bar{SD}_{w}$) quantified on the original data scale ($w_{ik}\pm\bar{SD}_{w}$) translates to a level of precision equal to ± 1 on the transformed scale ($y_{ik}\pm1$). The transformation operated can thus be summarized as follows:

$$w_{ik}\pm\bar{SD}_{w} \underset{\Rightarrow}{\times\frac{1}{\bar{SD}_{w}}} y_{ik}\pm1 .$$

Transformed data points are obtained as:
gnitude ofthe relevant level of precision to be kept from the original dataocess

$$y_{ik}=w_{ik}\times\frac{1}{\bar{SD}_{w}} ,$$

and the level of precision on the transformed scale is:

$$\bar{SD}_{y}=\bar{SD}_{w}\times\frac{1}{\bar{SD}_{w}}=1 .$$
